# From controllers to cognition: the importance of selection factors on video game and gameplay mechanic-derived cognitive differences

**DOI:** 10.1186/s12993-024-00258-7

**Published:** 2024-12-20

**Authors:** Tina T. Vo, Shandell Pahlen, Anqing Zheng, Sian Yu, Emery Lor, Nicholas D. Bowman, Robin P. Corley, Naomi P. Friedman, Sally J. Wadsworth, Chandra A. Reynolds

**Affiliations:** 1 Department of Psychology, University of California, Riverside, Riverside, CA 92521 USA; 2 Institute for Behavioral Genetics, University of Colorado Boulder, Boulder, CO USA; 3 Newhouse School of Public Communications, Syracuse University, Syracuse, USA; 4 Department of Psychology and Neuroscience, University of Colorado Boulder, Boulder, CO USA

## Abstract

Playing video games, especially games with action-based mechanics, is correlated with better cognitive performance, yet these performance advantages may originate from intrinsic factors such as earlier life cognitive differences. We investigated whether gaming-cognition associations in a sample past young adulthood remain robust after accounting for adolescent cognitive functioning. Using data from the Colorado Adoption/Twin Study of Lifespan behavioral development and cognitive aging (CATSLife; N = 1241, M_age_ = 33.3, %, age range = 28–51, Female = 52.9%), we compared cognitive performance of adult recreational gamers (40.6%) to non-gamers (59.4%) and between different types of gamers. Measures included processing speed, spatial reasoning, and working memory cognitive tasks, gaming status, and gameplay type engagement. The majority of gamer participants reported exclusively playing puzzle/strategy/life simulation games (53.0%) or action type games (33.1%); a smaller proportion reported playing both types of games (10.3%). Overall, gamers significantly outperformed non-gamers across most cognitive tasks (Cohen’s d = 0.17–0.25), with limited evidence of a differential gameplay mechanic effect across tasks. Selection effects were evident whereby after adolescent IQ adjustment, gamer cognitive effects diminished by over 35% but persisted for spatial performance. Adolescent IQ predicted puzzle/strategy/life simulation preference but not action-type games, suggesting a selection effect. Our study replicates prior gaming findings and reveals that earlier life functioning contributes to adult gaming-cognition associations. Gamer-spatial associations are not fully attributable to intrinsic factors, and playing video games, regardless of a specific gameplay mechanic or genre, may represent a cognitively engaging lifestyle behavior that may benefit cognitive functioning, with implications for preserved cognition.

**Supplementary Information:**

The online version contains supplementary material available at 10.1186/s12993-024-00258-7.

## Introduction

Video game play is commonly ranked among the top ten recreational activities in the United States (Statista, 2023), has shown fast-paced growth outside of the top 3 countries of China, the US and Japan, and has shown substantial gaming revenue increases in Africa, Latin America and Southeast Asia between 2017–2023 (Statista, 2024). Moreover, there are projected increases to 3.63 billion gamers worldwide by 2027 (Statista, 2024). People play video games to meet specific individual needs, including socialization/social connectedness, competition, and distraction from everyday life [59, 92]. Over the past 15 years, research has championed the exploration of gaming-cognition links, specifically the observed performance advantage gamers show on many specific cognitive abilities [8, 66]. Video games include a broad swath of game design and mechanics, which could have differential associations with cognitive performance [7, 55], or attract different types of players [6, 67]. Moreover, these correlated benefits may be due to reverse causation or selection effects [7, 16, 38]. Investigating the connection between gaming and cognitive performance past young adulthood while considering earlier life functioning represents an important step in understanding their interrelationship and saliency of this behavior on later cognitive aging.

Video game engagement is a common leisure activity and a lifestyle behavior that has been posited to be a viable cognitive health intervention tool for later cognitive decline or dysfunction [5, 79]. Cognitive aging decrements for many specific cognitive abilities, such as spatial reasoning, processing speed, and working memory, occur decades prior to midlife [42]. For these domains, researchers found that performance peaks during young adulthood up to early 30s [42] with expectant declines thereafter [50, 62]. The age period occurring between the end of emerging adulthood and up to midlife has been coined as ‘established adulthood’ [52], covering the ages of 30 up to 45 years. This age range is a salient time for cognitive aging and lifestyle behaviors that influence later health and functioning as individuals approach the midlife transition and traverse up to old age. Empirical studies have also highlighted that established adulthood is an under-researched life stage, especially in the context of cognitive research [93]. Moreover, this is a period marked by change in environmental demands (e.g., familial obligations, career, and occupational responsibilities, that may substantially affect how individuals select and allocate their time for leisure activities such as video gaming.

Contemporary video game play represents a merger of technology and leisure, with evolving complexity in design and mechanics that can incorporate similar play dynamics as real-world physical leisure activities. Just as in video games, play mechanics can vary on skill and game demands in sport activities, contributing to differential effects between physical activity and cognitive health [74]. Sport activities that are considered “Open-skill” (e.g., volleyball), which contrasts with “close-skill” (e.g., swimming), include sports games that place greater perceptual, attentional, and motor demands on players as they respond and adapt to dynamic and competitive play. Consequently, examining video gaming, a leisure activity that is increasingly growing in popularity, could capture this greater individual cognitive demand than other cognitive leisure activities, such as board games. For example, a study examining the difference in cognitive performance between video gamers and board gamers found that more time spent playing video games was associated with better performance on several specific abilities, including working memory and visuospatial processing [49]. Even though some of the first video games were based on board games (e.g. chess) and have continued to incorporate their mechanics, the challenge and depth of video game play may provide more cognitive benefits than the traditional analogue form.

Investigations of gaming and cognition have a history dating back over 35 years, with some of the first studies finding gamers’ better performance on spatial ability tasks [35]. This finding has continued to be supported in studies comparing gamers to novices [8] and training/intervention game studies [66, 82]. Spatial ability refers to the skill of mentally representing and manipulating objects within a space, and some games employ navigational and orientational mechanics that engage this ability [7, 75]. The observed higher specific cognitive performance gamers exhibit may arise from players exercising certain cognitive abilities to play the game well [16, 25]. For example, gamers who play action-oriented games that require making quick, complex decisions that are time-sensitive (e.g., target and offensive choices against an opponent in a multiplayer game) may practice and improve cognitive skills related to the speed of processing [47, 48], or the ability to quickly resolve problems [68]. Likewise, processing speed is posited to share dynamics with many types of gameplay mechanics, such as speed-based tasks that balance perceptual demands and accuracy [7, 8, 39]. Players also pursue game goals [7] while maintaining and processing actively relevant mental information [18]. Gamers have shown better performance on working memory tasks that engage this mechanic [4, 17].

Habitual video game players that play action games demonstrate small advantages on working memory tasks; however, these advantages are stronger in visuospatial rather than verbal domains [7, 8]. Specifically, video gamers showed better working memory performance on visuospatial variants of simple span, complex span, and running memory tasks compared to numerical-verbal variants of these tasks [87]. Enhanced verbal memory performance on digit span was found in a training study using the “brain game” Luminosity, but associations were stronger for digits forward, which is more reliant on attentional resources and more demanding on attentional control than digits backward [79]. Although engaging in video game play has shown evidence of enhanced cognitive skills, the effects are not ubiquitous across all games [16, 25] as cognitive demands (along with emotional, physical, and social demands) vary between video games [11].

Technological advances have enabled greater sophistication and complexity in game design since the arcade games of the past and led to the diversification of game types and mechanics [20]. Despite the desire and need to categorize and describe the diverse gameplay available to individuals, this remains a difficult and complex task. Indeed, consistent classification and operationalization of types of leisure activities has remained elusive with a variety of methods currently existing [29]. Nonetheless, categorizing video games, even in a broad sense, can be helpful to uncover differential effects between gamers for early investigations or studies that want to incorporate gaming in their research aims but lack detailed gaming history or qualitative reporting. Efforts to classify the vast number of games on the market have varied but common methods have employed genre categorization centered around shared gameplay mechanics, with the broad class of action genre being predominantly represented in the video game-cognitive literature [7, 8, 16, 73].

Games are often classified as an action video game [7] if they include the elements of (1) being fast-paced (e.g., requiring rapid motor responses such as quick reaction times to fast-moving objects), (2) requiring high cognitive load (e.g., requiring players to attend to multiple sources of stimuli and goal states), (3) include variable attentional demands (i.e., high-focus for aimed targets versus broad/distributed-focus for full field of view monitoring), and (4) include distractions (e.g., non-targets are distributed within the field to distract players from targets or objectives). Not all these elements need to be simultaneously and continuously present for a video game to be considered an action video game, but action video games will predominantly feature many of these core gameplay mechanics to satisfy the game goal or objective. Not only has research centered around action video games’ influence on cognitive performance due to the popularity of the action genre but also how these underlying specific mechanics may influence cognitive ability. When games include action components and fast-paced mechanics, greater demands are placed on attentional resources, perceptual speed, spatial abilities, and short-term memory [8, 21, 75].

**Fig. 1:**
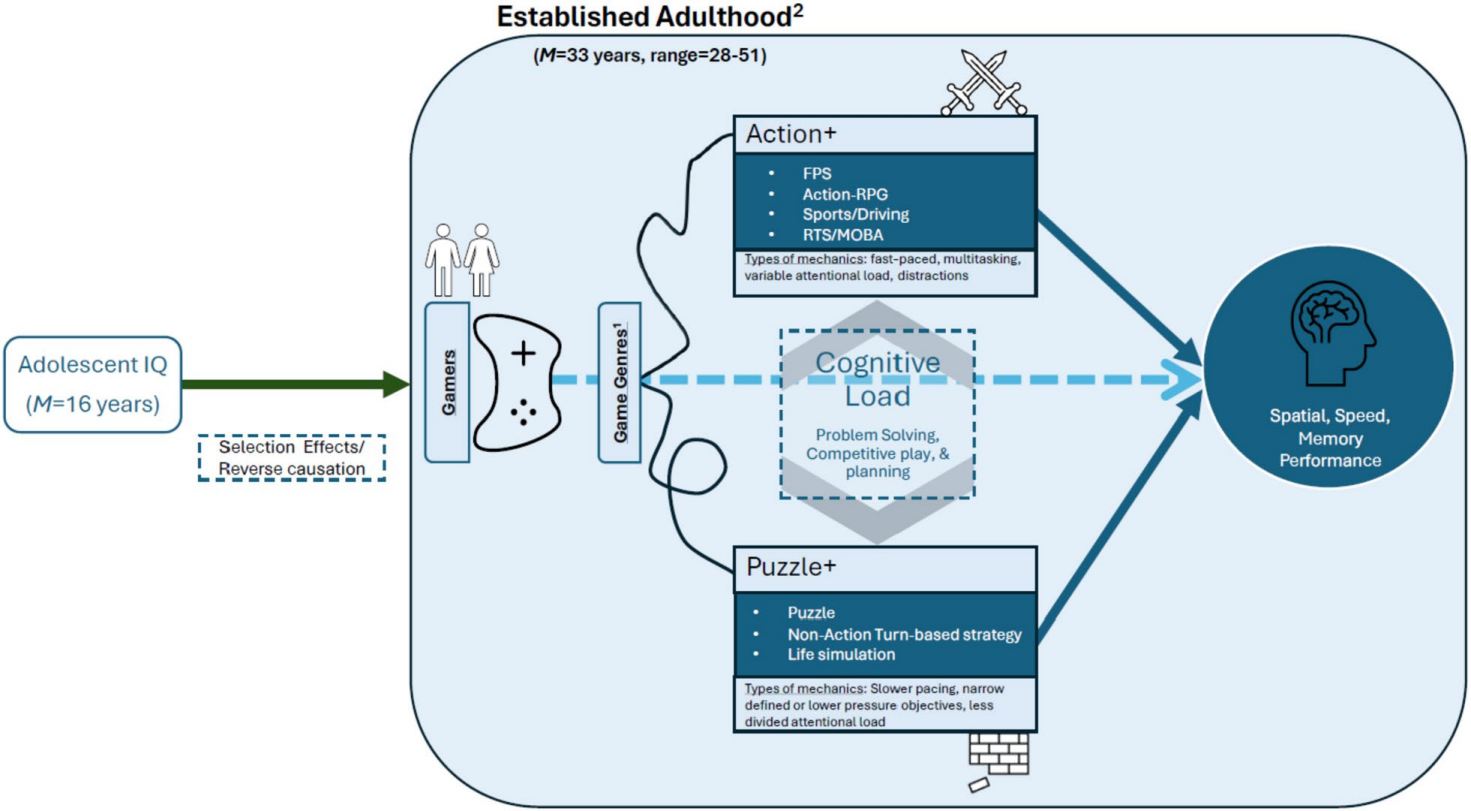
Conceptual model *FPS* = First/Third person shooters, *Action-RPG* = Action-Role Play Game, *RTS* = Real-Time Strategy, *MOBA* = Multiplayer Online Battle Arena (MOBA). ^1^[20], ^2^[52]

Although action represents a common research genre category, the action genre is a broad class of games including many subgenres such as first/third person shooters (FPS), action-adventure Role-playing game (RPG), sports/driving, and Action-Multiplayer Online Battle Arena (MOBA) and Action Real-Time Strategy (RTS) [20]. Popular action video games include Overwatch, Legend of Zelda, Witcher, and Mario Kart. There remains a debate on the specific subgenres, specific games, or the play time required to be categorized as action video games or individuals to be categorized as action gamers [20]. For example, MOBA (e.g. League of Legends) and RTS (e.g. Starcraft) video games have often been excluded from the action genre [7, 8, 22]. This subgenre exclusion represents a historical shift in game design and genre hybridization over the last 20 years, with many action components having been integrated in these types of games since their initial introduction, see Dale & Green,(2017) for further discussion. Research examining MOBA and RTS suggests cognitive advantages and engagement of many overlapping cognitive systems that are similar to purely FPS action games [3, 5], [22], [21, 36], thus indicating alignment of the specific cognitive demands of this subgenre with other action video games (see Fig. 1).

Several meta-analyses have supported the associative benefits of playing action-forward games, which we will refer to as Action + games, on specific cognitive abilities, including spatial ability and processing speed with less consistent evidence for working memory [8, 9, 66, 73, 86]. Nonetheless, Action + video games only represent a proportion of games that casual gamers may particularly gravitate towards. Specifically, puzzle/strategy/life simulation-type games [7, 8], which we will refer to as Puzzle + games, rival in popularity to the engagement in Action + games [88]—by some estimates, Puzzle + games are the most popular game type among broader populations [28].

Within current research, strict comparisons are often made between action and non-action genres, with the Puzzle + games often categorized as non-action [7, 8, 66, 73, 86]. Puzzle + games are defined broadly in contrast to the Action + games where, gameplay mechanics in Puzzle + games are typically slower or have more narrowly defined objectives. Some linking elements seen across Puzzle + games include (1) lower pressure task objectives (e.g., pattern recognition, sequence solving, social simulation), and (2) low divided attentional load, as compared to action genre [20]. Some popular Puzzle + game titles include, Bejeweled, Tetris, Puzzle, Civilization, and Hearthstone [8]. Given the prioritization of Action + games in the literature due to the theorized wider and more taxing cognitive load of action games across cognitive domains [75], research directly examining Puzzle + influence on cognitive performance has lagged behind. This is somewhat supported by associated cognitive benefits spanning several cognitive domains from playing Action + games [7, 8] as compared to Puzzle + games. Findings linking Puzzle + games to cognitive performance tend to be more mixed, potentially due to poorer representation across studies. This variability may be attributed to the focus of many studies on training with simpler Puzzle + games like Tetris [66] or comparisons involving training groups versus inactive control groups [57]. However, the connection between gamers who engage in Puzzle + game play and improved spatial ability performance is one of the more consistent findings [32, 58, 66]. Engagement with Puzzle + game play also intersects with demographic patterns in gaming preferences. Notably, females and older adults ––populations less represented in current literature [16] –– are more inclined towards puzzle and strategy games [6, 67]. A focus on Action + games could potentially overlook the cognitive effects of Puzzle + games in these demographic groups. Indeed, meta-analyses highlight the role of age in cognitive studies, suggesting that the cognitive benefits associated with Action + games are less consistent and otherwise lower in older adults compared to younger populations [7, 16, 66, 86]. For specific cognitive abilities, age differences are more prominent than sex differences [33], with younger adults outperforming older adults on many abilities [42]. With shifts in gaming demographics toward females and older gamers [28], research must consider the gaming mechanics that may be derived from certain video game genres these populations prefer to play.

Extant literature has demonstrated positive associations between gaming and cognitive functioning, with differential effects between types of players. Yet, gaming-cognition links may also be due to player background. Effects may arise from reverse causation, necessitating research to account for selection factors such as individual differences that may influence people to become gamers [9, 10]. For example, individuals who tend to be more adept cognitively may be more likely to be or become gamers, and the cognitive differences observed may arise from subject biases rather than recreational engagement in video games per se [7, 10, 16]. Reverse causation/selection effects are a major concern in lifestyle and cognitive health literature, particularly for the correlated links found among cognitively engaging activities [9] such as playing video games. It is possible that accounting for earlier life cognitive functioning fully attenuates video gaming-cognition connections, suggesting reverse causation. However, not all research indicates effects emerge due to intrinsic factors. Playing board/solitaire games was associated with higher cognitive performance and dampened decline among older adults, and these effects were only partially accounted for by childhood cognition [2]. Whereas prior video gaming studies have evaluated and accounted for underlying methodological concerns in the gaming-cognition literature, such as expectance/placebo effects, suggesting limited bias in current findings [7, 10], most studies do not or cannot account for earlier life functioning. Moreover, much cross-sectional research relies on convenience samples that tend to be male-dominated or younger/college-aged samples (e.g., [7, 16]). The differential cognitive links between game genres or comparison between types of players may operate as a function of individual differences influencing player preference, such that selection effects may be greater for a particular genre effect. In addition, players tend not to overlap between action or puzzle genre preference, which may be explained, in part, by the player motivations or challenge and mechanics of the genre [69].

While gaming may provide some cognitive performance benefits based on findings from correlational and intervention studies (e.g., [53, 57, 71]), many questions linger. We leverage data from the Colorado Adoption/Twin Study of Lifespan behavioral development and cognitive aging (CATSLife [85]) to further unpack gaming-cognition associations. Specifically, our study focuses on a broad cross-sectional community-based sample of adults in the established adulthood [52] period, which is underrepresented and under-explored in the existing literature. The unique and comprehensive data available in CATSLife also enables us to explore how earlier life cognition may influence the selection of video game engagement and engagement with differential game mechanics, potentially underlying gaming-cognition outcomes. As we outline in Fig. 1, our study objectives were twofold: (1) we first examined general video game play associations with specific cognitive abilities of processing speed, working memory, and spatial reasoning; (2) we further examined the relationship between specific gaming mechanics, derived from Action + and Puzzle + game play, with cognitive performance. Within both objectives, we account for selection features by including earlier life cognitive ability indexed by adolescent IQ scores to examine whether gaming-cognition associations are captured by earlier cognitive differences.

### Method

#### Participants

The current cross-sectional sample was derived from the first completed assessment of the CATSLife (CATSLife1; [85]) study, conducted between the years 2015 to 2021, with ongoing efforts for data collection in CATSLife2. The CATSLife1 sample is an independent sample that was derived from two longitudinal parent studies (i.e., the Colorado Adoption Project (CAP, c.f., [63, 64]) and Longitudinal Twin Study (LTS, [63]). Briefly, CAP and LTS projects followed similar protocols and recruitment schedules starting at birth and continuing through adolescence and early adulthood with the aim of understanding complex behavior and development across time but differed in family and sibling composition. Together, CAP and LTS encompass both adopted and non-adopted sibling pairs as well as same-sex twin pairs. The CATSLife1 sample includes 1,327 individuals ranging from 28 to 51 years (*M*age = 33.45 years, *SD* = 5.04, 53.1% female). The majority of participants were White (92.2%) and non-Hispanic (94.3%).

**Fig. 2:**
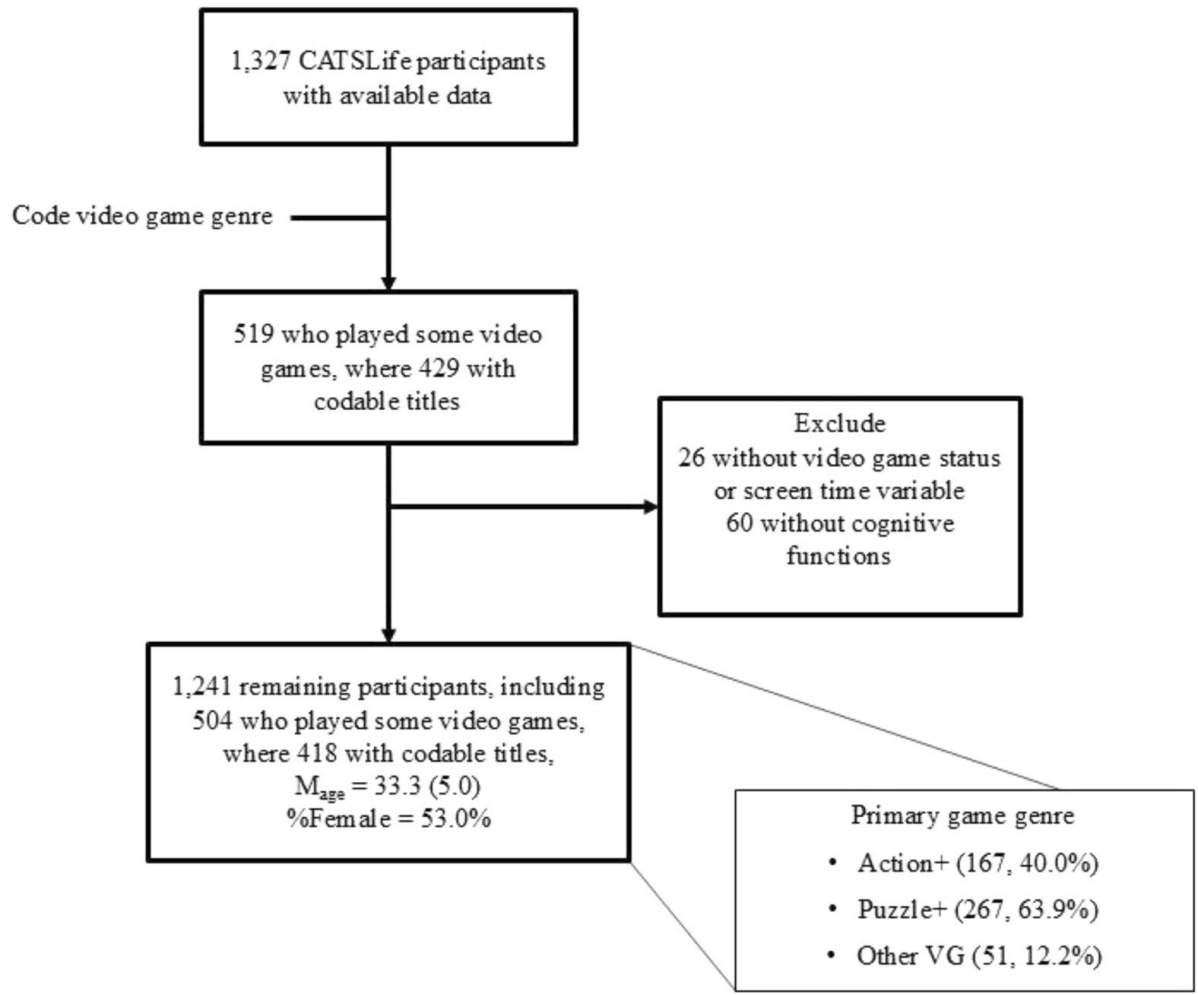
CATSLife Analytic Sample Composition

While the full CATSLife sample comprises 1327 individuals, the analytic sample consisted of 1241 (52.9% female) individuals ranging in age from 28–49 years (Mage = 33.3, SD = 5.0). This reduction in sample size was due to our inclusion criteria, which required participants to have scores for at least one cognitive outcome measure and have complete data for digital screen time measures, including video game play (see Fig. 2). Sensitivity analyses were conducted with a gamer-only subsample (N = 418, 49.3% female); sample descriptive statistics were comparable to the analytic sample (see Table 1).

**Table 1:** Demographic and sample descriptive statistics

	Measure	Analytic sample N = 1241	Gamer subsample N = 418	Non-gamers N = 823
Demographics	Age, M (SD)	33.3 (5.0)	34.0 (5.1)	32.9 (4.9)
	Female, %	52.9%	49.3%	58.5%
	White, %	92.0%	92.3%	91.7%
	Hispanic, %	5.7%	5.5%	6.0%
	Years of education, M (SD)	15.6 (2.1)	15.5 (2.1)	15.8 (2.2)
Cognitive measures	*Processing speed*			
	Digit symbol, M (SD)	85.8 (15.1)	86.7 (15.7)	85.3 (15.6)
	Colorado perceptual speed, M (SD)	39.6 (8.1)	40.3 (8.4)	39.1 (7.9)
	Subtraction and multiplication, M (SD)	31.1 (15.8)	33.0 (17.1)	30.3 (15.0)
	*Working Memory*			
	Digit span, M (SD)	18.1 (3.9)	18.3 (4.1)	18.0 (3.8)
	*Spatial reasoning*			
	Block design, M (SD)	51.1 (9.9)	52.4 (10.2)	50.1 (9.6)
	Paper Form Board, M (SD)	11.9 (4.0)	12.7 (4.4)	11.5 (3.7)
	Card Rotations, M (SD)	131.8 (38.8)	136.4 (39.1)	127.9 (37.8)
	Adolescent IQ, M (SD)	110.2 (11.8)	112.8 (12.2)	109.1 (11.2)
Screen time and gameplay measures	Digital screen time, M (SD)	4.3 (2.6)	4.9 (2.5)	3.8 (2.6)
	Gamer/non-gamers, N	504/737	418/0	0/823
	Action + gamers, N	167	167	0
	Puzzle + gamers, N	267	267	0
	Action + & Puzzle + gamers, N	52	52	0
	Other gamers, N	36	36	0

Analytic N for cognitive measures range: 1213–1241; gamer sample N = 410–418; non-gamer sample N = 718–737

### Measures

#### Cognitive measures

This study utilized seven cognitive measures spanning three cognitive domains: processing speed, working memory, and spatial reasoning. Reliability estimates across the seven cognitive measures ranged from 0.81 to 0.96 (see [23], [60]). Follow-up analyses included adolescent general cognitive ability indexed by IQ scores to examine how earlier life cognitive differences may influence video game-cognition associations. The specific cognitive measures by cognitive domain and adolescent cognition are described in detail below. Descriptive statistics for scores across all cognitive tasks are available in Table 1 for the analytic sample and the gamer subsample.

##### Processing speed

Processing speed was assessed using three assessments: Digit Symbol (Dsym) subtest from the Wechsler Adult Intelligence Scale- Third Edition (WAIS-III; [89]), the Colorado Perceptual Speed (CPS) task [23], and the Subtraction and Multiplication (SAM [27]) test. For Dsym, participants were tasked with reproducing the symbol to the corresponding paired digit. For CPS, participants were tasked with identifying a target letter string amongst distractors. Lastly, for SAM, participants were tasked with quickly and accurately answering multiplication and subtraction problems within a limited time frame (i.e., 4 minutes).

##### Working memory

Working memory was assessed through Digit Span (Dsp; [70]). Digit Span is the total score correct where participants accurately repeated increasing spans of numerical digits in either the presented order or in the reverse order.

##### Spatial reasoning

Spatial reasoning was assessed using three tasks: Block Design subset (BD) from the WAIS-III [89], the Paper Form Board (PFB) subtest from the Hawaii Family Study of Cognition (HFSC [24]), and the Card Rotations (CR) subtest from the Educational Testing Service (ETS [27]). For BD, participants were tasked with quickly and accurately matching a target 2-dimensional pattern with 3-dimensional blocks. For PFB, participants were tasked with reproducing target shapes by selecting only the correct cut lines in the tested shape. For CR, participants were tasked with deciding whether eight shapes matched the same side or the opposite side of a target shape under various rotations.

##### Adolescent IQ

Adolescent general cognitive ability was derived from full-scale WAIS IQ measures (WAIS-R or WAIS-III; [89, 90]) assessed approximately at age 16. On average, participants completed the year 16 assessment 16.72 (*SD* = 5.02) years prior to CATSLife. After further review of the genre effects we observed, we included adolescent Block Design derived from the WAIS-III Block Design subtest assessed at the same wave for a post-hoc genre analysis. Scores for the WAIS-R version of Block Design were rescaled to WAIS-III scores through the percent correct method. Adolescent Block Design was mean centered at 48.

### Digital screen time

Participants reported their digital screen time from a single item derived from a 20-item activity engagement scale adapted from Jessor and Jessor [44]: “*About how many hours do you usually spend each week using a computer, smart phone, tablet, or video game machine just for fun*?”. Participants reported hours ranging from “none” to “8 or more hours a week” and responses were rescaled to hour increments, averaged across hours: “None” = “0”, “1 hour” = “1”, “2–3 hours” = “2.5”, “4–5 hours” = “4.5”, “6–7 hours” = “6.5”, and “8 or more hours” = “8”. Separate inventory items specifically asked about time watching television and were not factored in our digit screen time measure. On average, CATSLife participants tend to spend approximately 4.3 hours (SD = 2.6) using digital devices, which was slightly less time compared to the gamer subsample (M = 4.9 hours, SD = 2.5), see Table 1.

### Video game play (Vgplay)

Video game players (n = 504) were identified if they reported any digital screen time and listed at least one video game title. Of note, a proportion of open-ended responses (n = 86) were considered vague and had no discernable video game title but clearly stated the participant plays video games by indicating a gaming console or providing a vague response (e.g., “XBox”, “Computer games”). Those cases were coded as “1” on VGplay, an endorsement of playing video games, but were not given a genre code as a specific genre was not discernable. Participants were considered non-gamers (n = 737) if they did not report any digital screen time hours per week or reported at least 1 h of digital screen time but only listed non-game apps or websites (e.g., “Facebook”).

### Game genre classification

Participants provided game titles they play in an open-ended response (e.g., “*What computer/smart phone apps/video games do you play?*”) if they indicated any digital screen time. Each game title was genre-coded according to one of the seven game genre categories identified in the Video Game Questionnaire [20]^1^: (1) First/Third person shooters (FPS), (2) Action-Role Play Game (RPG)/Adventure, (3) Sports/Driving, (4) Real-time Strategy/Multiple Online Battle Arena (MOBA), (5) Turn-Based/Non-action RPG/Fantasy; (6) Turn-based Strategy/Life simulation/Puzzle, or (7) Music. We created an eighth category of “Other” if the game title did not fit into a specific genre (e.g., phone games, fighting games, brain/language games). Briefly, game genre classification was conducted using a blind double-entry method, where coders demonstrated moderate reliability to each other (k = 0.74, 95% CI [0.70, 0.78). A consensus meeting reviewed all genre codes and resolved coders’ disagreements and assigned final genre codes. Consensus members were lab faculty and graduate students and did not include any original coders. Final genre codes were informed by coders’ familiarity ratings along with consensus members’ own familiarity and game review. Comparing original coders and consensus coders, genre coding reached good levels of reliability (k = 0.85, 95% CI [0.83, 0.88]), suggesting general agreement of genre classification between initial coders and consensus coders. Overall, 418 participants were identified as playing at least one genre; descriptive statistics for genre frequency are provided in Table 1. Relevant to our research aims, we prioritized examining cognitive performance differences comparing players based on differential gameplay mechanic influences that may derive from Action + and Puzzle + game play.

*Action +* These games generally have gameplay mechanics related to being fast paced, which require divided attention, switching focus between different gameplay demands, and variability in mechanics where performance cannot rely on simple habitual actions [20]. Subgenres of action games include first-person shooters (FPS,e.g., *Call of Duty*), action-adventure/roleplaying games (action-RPG; e.g., *The Legend of Zelda: Breath of the Wild*), sports/driving simulations (e.g*., Need for Speed*), and real-time strategy games (e.g., *StarCraft*). Action + gamers (n = 167) were dichotomously coded comparing those playing at least one Action + game (1) or “other” (0). “Other”, in this case, referred to any other discernible game titles that did not meet the criteria of being an Action + game.

*Puzzle + * These games generally emphasize gameplay mechanics related to deliberate decision-making, logical challenges, and turn-based rather than real-time play. Puzzle + games usually include turn-based strategy games (e.g., *Civilization*), life simulation/virtual world games (e.g., *The Sims*), and classic puzzle games (e.g., sudoku or jewel match games). Notably, puzzle, strategy, and life simulation games share some overlapping gameplay mechanics, including problem-solving, resource management, strategic planning, incremental progression, decision- making, pattern recognition, and goal-oriented tasks. Some puzzle games even simulate real-world conditions, such as real-world physics. In contrast to Action + games’ speed and divided attention requirements, Puzzle + games allow time for deliberate thought before acting. Puzzle + gamers (n = 267) were dichotomously coded comparing those playing at least one puzzle + game (1) or other (0). “Other”, in this case, referred to any other discernible game titles that did not meet the criteria of being a Puzzle + game. Of note, exclusive mobile games like “Candy Crush” are excluded from the Puzzle + games, because of their advertising disruptions, and the free-to-play progression barriers tied to in-app purchases commonly found in mobile games but not in traditional computer and console games.

### Contrast codes comparing differential gameplay mechanics

We developed three contrast codes following the procedure proposed by Yaremych and colleagues [91] to examine and compare differential gameplay mechanic-specific effects. As the evaluation of the different type of gamers were the primary focus, across all three contrast codes, non-gamers or those missing on a video game title were assigned a value of “0”.

*Action + or Puzzle + vs Other contrast (Other)* This contrast probes the effect of Action + , Puzzle + , or cross-play gamers vs. other players. Players of video games other than Action + or Puzzle + (n = 36) received a “−0.75” score, while those playing Action + , Puzzle + , or a combination of both (n = 382) were scored “0.25”. As such, lower scores indicate other gamers and higher scores indicate Action + or Puzzle + gamers.

*Cross-play contrast (Cross)* This contrast probes the effect of playing exclusively one gameplay mechanic (i.e., Action + or Puzzle +) vs. the potential benefits of cross-genre play (i.e., Action + and Puzzle +). Those playing exclusively Action + or Puzzle + (total n = 330) received a score of ‘-0.33’, while those playing both (n = 52) were scored ‘0.667’. As such, lower scores indicate engagement with a single core gameplay mechanic and higher scores indicate cross-play Action + and Puzzle + gamers.

*Action + only vs. Puzzle + only contrast (Gameplay Mechanic)* This contrast compares the effect of engagement in Action + vs. Puzzle + games. Specifically, Puzzle + gamers (n = 215) are assigned a “-0.5” and Action + gamers (n = 115) are assigned a “0.5”. Players that indicated playing both were assigned a “0”. As such, lower scores indicate Puzzle + gamers while higher scores indicate Action + gamers.

### Educational attainment (ISCED)

Participants self-reported their educational attainment from 10 given categories, spanning from obtaining less than a high school diploma or GED, to possessing an advanced degree such as a doctorate, M.D., or law degree. These classifications were then aligned with the International Standard Classification of Education (ISCED; [80]) according to the classification that best fit within the 7 distinct levels of educational completion outlined in the ISCED framework. ISCED was centered at 16 years, equivalent to attaining a bachelor’s degree.

### Covariates

Covariates known to be associated with specific cognitive performance, such as age, sex, ethnicity, race, and educational attainment, were included [1]. To maintain model parsimony, socioeconomic status (SES) considerations were limited to the individual’s educational attainment. Additionally, adjustments were made for cohort differences between participants from CAP or LTS, with LTS serving as the reference group. Adoption status was also included as a covariate, given that prior work from CAP has found developmental cognitive differences emerge between adopted and non-adopted individuals [65]. Overall, our covariate adjustment accounted for well-known factors associated with cognitive functioning. The covariates included were age (centered on the mean: 33 years), sex (0 = Female, 1 = Male), project (0 = LTS, 1 = CAP), adoption status (0 = Non-adopted, 1 = Adopted), ethnicity (0 = Non-Hispanic, 1 = Hispanic), race (0 = Non-White, 1 = White), educational attainment (centered at 16 years), and digital screen time (centered at 1 h). To adjust for the elapsed years between the age 16 and CATSLife assessments, we entered the time difference in years (centered at 15) between the CATSLife and the year 16 assessment when adolescent IQ was included in the analyses. This adjustment for the elapsed years was included since there was a wider age range for the age 16 battery for the CAP participants.

## Statistical analyses

All analyses were conducted in SAS version 9.4 (SAS Institute, Cary, NC). We employed generalized estimating equations (GEE; [41]) using the PROC GEE procedure in SAS to account for the correlated structure of sibling data within CATSLife. This approach corrects the standard errors of the regression parameters for the non-independence of the family-level clustered data, effectively addressing intra-cluster correlation [51, 54]. All models accounted for age, sex, project, adoption status, race, ethnicity, educational attainment, and digital screen time. For later models, we included adolescent IQ, and years follow-up between the year 16 assessment and CATSLife. Our analytic plan was two-fold: (1) to examine general VGplay associations with each specific cognitive ability and (2) to further investigate the relationship between specific gameplay mechanics that stem from genre categories (i.e., Action + and Puzzle +) with cognitive performance. To check the robustness of our findings and account for selection, we included adolescent IQ to check whether gaming-cognition associations remain consistent after considering earlier life cognitive function. As a sensitivity check, we then repeated analyses with a gamer-only sample to test whether specific Action + or Puzzle + advantages on cognitive performance emerged or persisted against other gamers.

### Video game play associations with cognitive performance

Our first research aim explored whether playing video games (VGplay = 1) is associated with better cognitive performance. This initial model was conducted for each cognitive task, including the effect of gaming while controlling for covariates. Parameter estimates for VGplay and digital screen time can be seen in Table 3 under VGplay only. All model parameters, including covariates, are available in Supplemental Tables ST 1–7.

### Video gameplay mechanic associations with cognitive performance

Our next research aim examined if engagement with a certain gameplay mechanic stemming from Action + , Puzzle + , or a combination of game play, made an additional contribution to cognitive performance. All models retained VGplay and sociodemographic covariates, differential gameplay mechanic effects parameter estimates for each cognitive measure are available in Supplemental Tables ST 1–7. We examined the differential gameplay mechanic effect by three group comparisons of gameplay mechanic, cross-play, and other contrasts. Once again, the “gameplay mechanic” contrast code compared Action + versus Puzzle + game play, with higher scores assigned to those who endorse Action + VGplay and lower scores assigned to those who endorse Puzzle + VGplay. The “cross-play” contrast code probes the effect of playing either Action + , or Puzzle + in comparison to playing both. As such, those playing exclusively one (either Action + or Puzzle +) were assigned lower scores while those playing both were assigned higher scores. Lastly, the “other” contrast probes the effect of playing either Action + , Puzzle + , or both in comparison to playing other genres where lower scores were assigned for those who play other genres. Of note, false discovery rate (FDR) corrections for multiple testing across cognitive tasks were done separately for the three gameplay mechanic-contrast indicators. We then examined if gameplay mechanic effects varied by digital screen time. Our last model represented a simplified approach concentrating on exploring the direct effect of gameplay mechanic comparison separately from the other contrast indicators. Selection effects were tested by including adolescent IQ and the difference in years between assessments for all models tested apart from gameplay mechanic-digital screen time moderation as we found little evidence of interaction between gameplay mechanic-cognition associations. Post-hoc analysis was conducted for Block Design which was the only task that evidenced specific Action + associations. To account for earlier life spatial ability, we included adolescent Block Design performance adjustment.

### Sensitivity analysis: genre-cognitive associations among gamers

To check the robustness and consistency of our findings, we concentrated and repeated analyses with only gamers to compare gameplay mechanic influences on cognitive performance. As only gamers were included, sensitivity analyses differed from the main analyses in that VGplay was not tested but rather the specific effect of Action + or Puzzle + compared to all other gameplay mechanics was tested. As such, the following three models tested the specific gameplay mechanic effect of Action + or Puzzle + , before and after accounting for adolescent IQ, as well as gameplay mechanic-digital screen time moderation. Sensitivity analyses followed the same procedure as the analytic strategy except gameplay mechanic differences among gamers were only examined with cognitive abilities that were significantly positively correlated (see Table 2). Thus, Puzzle + associations were only tested with processing speed tasks of Dsym and CPS (Supplemental Tables ST 8 & 9) and Action + with spatial reasoning tasks of BD and CR (Supplemental Tables ST 10 & 11). We further explored whether early IQ predicted later video game preference (i.e., Action + or Puzzle +). Within these logistic mixed-effects models, Action + or Puzzle + VGplay was the primary binary outcome and included the effect of adolescent IQ while controlling for covariates (Supplemental Table ST 12).

**Table 2:** Correlations

	Processing speed			Working memory	Spatial reasoning		
Measure	Dsym N = 1213	CPS N = 1241	SAM N = 1233	Dsp N = 1214	BD N = 1214	PFB N = 1240	CR N = 1222
Age	−0.04	−**0.08**	**0.22**	**0.09**	−0.02	**0.11**	0.03
Male	−**0.22**	−**0.09**	−0.02	0.03	**0.15**	**0.11**	**0.19**
White	0.05	0.02	0.02	0.05	**0.07**	0.04	**0.06**
Hispanic	−0.03	0.00	−0.06	−**0.06**	−0.04	−0.03	−0.06
DST	0.00	0.01	0.02	−0.02	0.03	0.02	0.03
Vgplay	0.04	**0.06**	**0.06**	0.02	**0.12**	**0.13**	**0.12**
Years of Educations	0.36	**0.32**	**0.31**	0.44	**0.22**	**0.14**	**0.13**
Action + Gamers	−**0.12**	−0.01	−0.03	0.06	**0.21**	0.08	**0.11**
Puzzle + Gamers	**0.19**	**0.12**	0.04	0.07	−0.07	0.00	−0.03

*Dsym* = Digit Symbol, *CPS* = Colorado Perceptual Speed, *SAM* = Subtraction and Multiplication, *Dsp* = Digit Span, *BD* = Block Design, *PFB* = Paper Form Board, *CR* = Card Rotations. Bolded parameters indicate *p* < .05.

## Results

### Correlations

Descriptive statistics for key variables are reported in Table 1. Unadjusted correlations among covariates, screentime, and gaming behavior with cognitive measures are reported in Table 2. We found that being a gamer (VGplay) was positively associated with most cognitive tasks spanning processing speed and spatial ability (*r*’s = 0.06–0.13, *p*’s < 0.05), where individuals who were gamers tended to perform better on cognitive tasks related to processing speed and spatial ability. While these associations are statistically significant (*p*’s < 0.05), the effect sizes are modest, suggesting that gaming is likely one of many factors influencing cognitive performance, and the effects should be considered in the broader context of other contributing variables (e.g., specific gaming genres, adolescent IQ, selection features). Differential gameplay mechanic effects were larger, compared to VGplay effects, and were observed between cognitive domains where being an Action + gamer was associated with spatial reasoning tasks (*r*BD = 0.21, p < 0.0001; *r*CR = 0.11, p < 0.05) and being a Puzzle + gamer was associated with better processing speed (*r*Dsym = 0.19, *p* = 0.0001; *r*CPS = 0.12, *p* = 0.02). For only one processing speed task was Action + gamers inversely correlated where Action + was associated with worse performance (*r*Dsym = -0.12, *p* = 0.01).

### Non-Gamer vs. gamer: cognitive performance

We evaluated general gaming associations with cognition by comparing performance between non-gamers and gamers (i.e., VGplay = 1). Table 3 reports gamer parameter estimates before and after the adjustment of adolescent IQ. After accounting for covariates, we found that being a gamer was significantly associated with better cognitive performance for nearly all cognitive tasks (all *p* ≤ 0.001), apart from working memory (B_Dsp_ = 0.27, *p* = 0.28). VGplay associations with cognitive performance were generally modest and consistent in effect between tasks (Cohen’s *d* = 0.17–0.25), with gamers tending to score a fifth of a standard deviation higher in BD than non-gamers.^2^ Adjusted VGplay unstandardized parameters diminished by half in estimate (35 to 54%) and effect size (Cohen’s *d* = 0.10–0.14), after inclusion of adolescent IQ controlling for elapsed years between assessments. These attenuation patterns would be suggestive of selection factors related to earlier life cognitive function underlying gaming-cognition associations. However, not all associations were lost. VGplay effects were reduced most for processing speed tasks, with only the association with CPS remaining significant (B = 1.10, *p* = 0.02) whereas all three spatial reasoning task effects remained robust (all *p* < 0.05*)*.

**Table 3:** Parameter estimates for gaming behavior

*Processing Speed*	Dsym N = 1164		CPS N = 1189		SAM N = 1181	
	Unadj	Adj	Unadj	Adj	Unadj	Adj
DST	0.03 [−0.28, 0.34]	−0.13 [−0.42, 0.16]	0.05 [−0.13, 0.23]	−0.03 [−0.2, 0.13]	0.02 [−0.32, 0.37]	−0.10 [−0.42, 0.23]
Vgplay	**2.75 [0.96, 4.55]**	1.49 [−0.2, 3.18]	**1.80 [0.81, 2.80]**	**1.10 [0.16, 2.04]**	**2.64 [0.72, 4.56]**	1.72 [−0.12, 3.56]
Adolescent IQ		**0.52 [0.44, 0.60]**		**0.30 [0.26, 0.34]**		**0.44 [0.36, 0.53]**

*Working memory*	DSp N = 1165					
	Unadj	Adj				
DST	−0.04 [−0.13, 0.05]	−0.08 [−0.16, 0.01]				
Vgplay	0.27 [−0.22, 0.75]	−0.06 [−0.52, 0.41]				
Adolescent IQ		**0.13 [0.11, 0.16]**				

*Spatial reasoning*	BD N = 1165		PFB N = 1188		CR N = 1171	
	Unadj	Adj	Unadj	Adj	Unadj	Adj
DST	−0.04 [−0.26, 0.18]	−**0.19 [**−**0.37, **−**0.01]**	−0.05 [−0.14, 0.04]	−**0.10 [**−**0.18, **−**0.02]**	−0.19 [−1.01, 0.64]	−0.50 [−1.27, 0.28]
Vgplay	**2.32 [1.13, 3.50]**	**1.07 [0.10, 2.04]**	**1.00 [0.51, 1.50]**	**0.54 [0.11, 0.97]**	**7.98 [3.30, 12.66]**	**4.46 [0.001, 8.92]**
Adolescent IQ		**0.5 [0.45, 0.54]**		**0.18 [0.16, 0.19]**		**1.10 [0.9, 1.31]**

Adj=adjusts for adolescent IQ and years-follow up. Vgplay=video gameplay, DST=digital screen time, Dsym=Digit Symbol, CPS=Colorado Perceptual Speed, SAM=Subtraction and Multiplication, Dsp=Digit Span, BD=Block Design, PFB=Paper Form Board, CR=Card Rotations. Bolded parameters indicate p < .05.

**Fig. 3:**
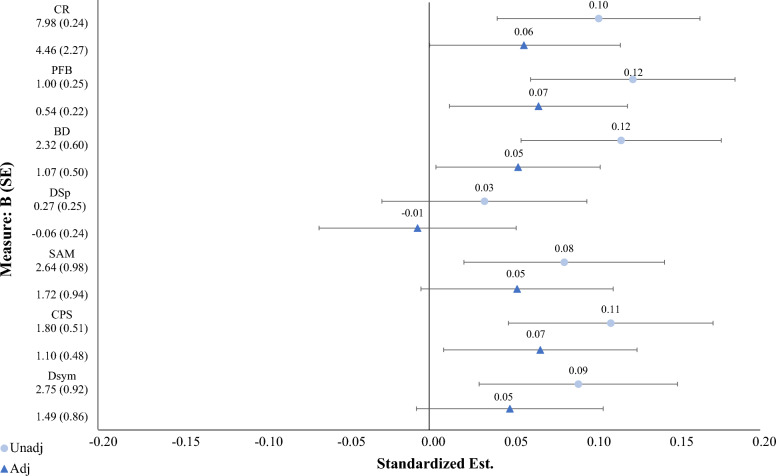
Standardized Parameter Estimates for Gamer Associations across Cognitive Tasks and Reduction in Effect Attributable to Adolescent IQ. *Unadj* = Unadjusted standardized parameter estimates without the inclusion of adolescent IQ, *Adj* = Adjusted standardized parameter estimates including adolescent IQ, controlling for elapsed years between assessments, *CR* = Card Rotations, *PFB* = Paper Form Board, *BD* = Block Design, *Dspan* = Digit Span, *SAM* = Subtraction and Multiplication, *CPS* = Colorado Perceptual Speed, *Dsym* = Digit Symbol

Gamer associations and the reduction in effect attributable to adolescent IQ are illustrated in Fig. 3 for all cognitive tasks. Estimated cognitive differences were significant between gamers and non-gamers. For example, gamers tended to score approximately 2.8 more points than non-gamers on Dsym. This difference between gaming groups leveled out after adjusting for adolescent IQ as seen in the reduction in predicted performance post-adjustment. After adjustment, gamers did score about 1.5 more points in Dsym but were not significantly outperforming non-gamers.

### Gameplay mechanic associations with cognitive performance

We evaluated whether differential effects emerged among gamers based on the gameplay mechanics most representative of their reported video game title and genre and whether these associations varied by digital screen time. Gameplay mechanic contrast parameter estimates comparing cognitive performance between exclusive Action + and Puzzle + players before and after adolescent IQ adjustment are reported in Table 4. We concentrate on reporting the gameplay mechanic contrast as we did not find strong evidence for cross-play or other genre direct effects after considering FDR correction for multiple testing or adolescent IQ adjustment (see Model 1 in Supplemental Tables ST 1–7). Likewise, we did not find evidence that gameplay mechanic associations varied by digital screen time; thus, moderation parameters are reported in Model 3 of Supplemental Tables ST 1–7.

**Table 4:** Parameter estimates for gaming and genre among total sample

*Processing Speed*	Dsym N = 1164		CPS N = 1189		SAM N = 1181	
	Unadj	Adj	Unadj	Adj	Unadj	Adj
DST	0.04 [−0.28, 0.35]	−0.13 [−0.42, 0.17]	0.05 [−0.13, 0.23]	−0.03 [−0.2, 0.13]	0.02 [−0.32, 0.37]	−0.1 [−0.43, 0.23]
Vgplay	**2.51 [0.59, 4.43]**	1.32 [−0.47, 3.11]	**1.75 [0.7, 2.79]**	**1.09 [0.1, 2.07]**	**2.61 [0.61, 4.61]**	1.76 [−0.15, 3.66]
Gameplay Mechanic Contrast	−2.00 [−5.74, 1.74]	−1.43 [−4.79, 1.93]	−0.46 [−2.45, 1.54]	−0.14 [−1.94, 1.66]	−0.24 [−4.27, 3.79]	0.32 [−3.43, 4.08]
Adolescent IQ		**0.52 [0.43, 0.6]**		**0.3 [0.26, 0.34]**		**0.44 [0.36, 0.53]**

*Working Memory*	DSp N = 1165					
	Unadj	Adj				
DST	−0.04 [−0.13, 0.05]	−0.08 [−0.16, 0.01]				
Vgplay	0.23 [−0.28, 0.74]	−0.06 [−0.55, 0.43]				
Gameplay Mechanic Contrast	−0.07 [−1.05, 0.92]	−0.04 [−0.98, 0.9]				
Adolescent IQ		**0.13 [0.11, 0.16]**				

*Spatial Reasoning*	BD N = 1165		PFB N = 1188		CR N = 1171	
	Unadj	Adj	Unadj	Adj	Unadj	Adj
DST	−0.05 [−0.27, 0.17]	−**0.20[**−**0.38, **−**0.02]**	−0.05 [−0.14, 0.04]	−**0.1 [**−**0.18, **−**0.02]**	−0.19 [−1.01, 0.64]	−0.5 [−1.28, 0.28]
Vgplay	**2.55 [1.34, 3.76]**	**1.37 [0.37, 2.37]**	**1.01 [0.5, 1.51]**	**0.57 [0.12, 1.01]**	**8.07 [3.2, 12.94]**	**4.66 [0.04, 9.29]**
Gameplay Mechanic Contrast	1.93 [−0.39, 4.26]	**2.48 [0.58, 4.38]**	0.04 [−0.97, 1.05]	0.23 [−0.65, 1.1]	0.74 [−8.38, 9.86]	1.7 [−6.77, 10.17]
Adolescent IQ		**0.50 [0.46, 0.54]**		**0.18 [0.16, 0.19]**		**1.11 [0.9, 1.31]**

*Adj* = adjusts for adolescent IQ and years-follow up. *Vgplay* = video gameplay, DST = digital screen time, *Dsym* = Digit Symbol, *CPS* = Colorado Perceptual Speed, *SAM* = Subtraction and Multiplication, *Dsp* = Digit Span, *BD* = Block Design, *PFB* = Paper Form Board, *CR* = Card Rotations, Gameplay Mechanic Contrast = compares Action + versus Puzzle + game play where Puzzle + gamers are assigned a “-.5” and Action + gamers are assigned a “.5”, and players indicating both genres are assigned a “0”. Lower scores indicate Puzzle + gamers while higher scores indicate Action + gamers. Bolded parameters indicate *p* < .05.

Across the cognitive domains, gameplay mechanic differential effects did not alter gamer associations seen previously. Overall, we did not find consistent evidence of a particular gameplay mechanic advantage for gamers after accounting for sociodemographic factors and digital screen time. The specific direction of the observed gameplay mechanic parameters should also be interpreted with caution due to the overlapping confidence intervals across cognitive tasks. For processing speed and working memory tasks, there was a nonsignificant performance advantage of Puzzle + compared to Action + gamers as indicated by the negative gameplay mechanic contrast coefficient. In contrast, spatial ability tasks demonstrated a nonsignificant Action + gamer advantage before accounting for adolescent IQ. After adjusting for adolescent IQ, however, associations were strengthened but only reached significance for BD (B_unadj_ = 1.93, *p* = 0.10; B_adj_ = 2.48, *p* = 0.01), suggesting exclusive Action + players tend to score a fourth of a standard deviation higher in the BD performance compared to Puzzle + players (Cohen’s *d* = 0.25). The enhancement of the Action + effect is suggestive of selection effects related to higher earlier life IQ and Puzzle + player’s gameplay mechanic preference. Given the exclusive gameplay mechanic associations found with BD, we assessed the persistence of effects after adjusting for adolescent spatial ability on the Block Design subtest. Post-adolescent BD adjustment attenuated VGplay B_unadj_ = 2.58, *p* < 0.001; B_adj_ = 0.75, *p* = 0.07) and the gameplay mechanic effect (B_unadj_ = 1.85, *p* = 0.11; B_adj_ = 0.35, *p* = 0.65), see Supplemental Table ST 5. Predicted performance in BD after adolescent BD adjustment is illustrated in Supplemental Figure S1.

**Fig. 4:**
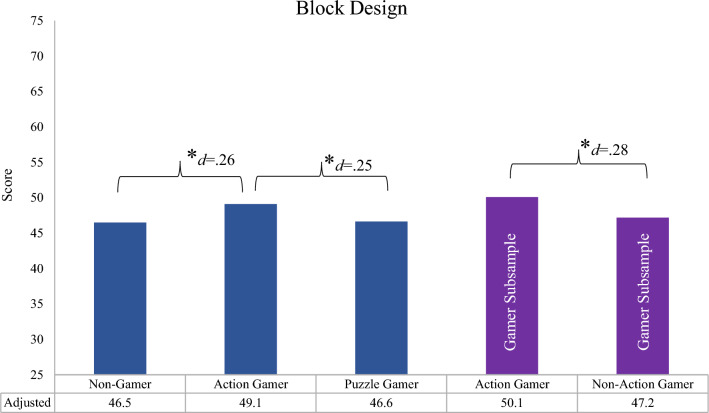
Block Design Gamer Associations and Effects Attributable to Adolescent IQ. Adjusted parameter estimates for Block Design scores including adolescent IQ adjustment, controlling for elapsed years between assessments. Purple bars indicate the gamer subsample

Predicted performance in BD for non-gamers and specific gameplay mechanic players after adolescent IQ adjustment are illustrated in Fig. 4. Estimated cognitive differences were significant between non-gamers and Action + gamers, with Action + gamers on average scoring approximately 3.5 more points than non-gamers on BD, which shrinks to 2.6-point difference after adolescent IQ adjustment. Action + gamers had a small advantage over Puzzle + players of 2 points in performance, which was non-significant. After adjustment, Puzzle + players’ performance is essentially equivalent to non-gamers; Action + gamers significantly outperform Puzzle + gamers by 2.5 more points in BD.

### *Sensitivity analyses: gameplay mechanic associations (Action* + *or Puzzle* +*) with cognitive performance among gamers*

We conducted sensitivity analyses assessing specific gameplay mechanic effects within a gamer only subsample (see Supplemental Tables ST 8–12). The selected gameplay mechanic-cognitive effects we examined were informed from bivariate associations indicating specific gameplay mechanic-performance benefits (see Table 2). As before, we found advantageous performance between Puzzle + players on processing speed and Action + players on spatial ability tasks; effects were nearly twice the size of those of the main analyses. Significant gameplay mechanic effects were observed for Dsym (B_puzzle_ = 3.8, *p* = 0.03) and BD (B_action_ = 3.68, *p* = 0.001) before adolescent IQ and years follow-up adjustment. Our findings suggest, compared to all other video gameplay mechanics, players who ever report playing Action + or Puzzle + games score approximately a fourth to over a third of standard deviation higher on Dsym (Cohen’s *d* = 0.24) and BD (Cohen’s *d* = 0.36), respectively. Post-adolescent IQ adjustment, we saw similar attenuation patterns in processing speed tasks as the main analyses; however, reduction in effects was greater by diminishing associations over 46%. Supplemental Figure S2 depicts the predicted Dsym performance for the gamer-only subsample in the purple bars separately for Puzzle + gamers and players of all others for pre- and post-adolescent IQ adjustment. Action + associations with BD performance remained robust (Cohen’s d = 0.28), after adjustment for adolescent IQ, however attenuation patterns differed from the main analyses. Across spatial ability tasks, with effects diminishing by over 21% as opposed to the strengthening of associations as seen in the main analyses. The gamer-only subsample’s predicted BD performance between Action + and all others after adolescent IQ adjustment is illustrated in Fig. 4.

Consistent with the main analyses, only Action + effects persisted for BD after accounting for earlier life cognition, and we did not find evidence that gameplay mechanic-cognition associations varied by digital screen time. Nonetheless, there were important differences. The greater gameplay mechanic effect seen in the sensitivity analyses was partially attributable to the categorization of Action + or Puzzle + players, where the inclusion of cross-players (N = 52) was included in both Action + and Puzzle + analyses and tended to be positively correlated with better performance across all cognitive tasks (see Model 1 in Supplemental Tables ST 1–7). Moreover, sex was predictive of preference for specific video gameplay mechanics (see Table ST 12) with males being over 11 times more likely to engage in Action + video games (OR = 11.47, 95% CI [7.03, 18.86]), and females being 8 times more likely to favor the Puzzle + video games (OR = 0.12, 95% CI [0.08, 0.21]). Additionally, higher adolescent IQ is associated with an increased log-odds of playing Puzzle + video games (0.02, 95% CI [0.001, 0.04]) but not Action + video games (0.01, 95% CI [-0.01, 0.03]). Together, even though unadjusted gameplay mechanic-cognition effects were stronger in the gamer-only subsample so too were the selection effects related to earlier life cognitive ability, particularly for those who ever reported playing Puzzle + games.

## Discussion

We explored the associations of gaming and differential gameplay mechanics with cognitive performance in an established adulthood sample. This CATSLife study reflects a large-scale effort to explore the nature of these associations in a sample with measures of earlier life functioning commonly unavailable in other studies. In general, we found that gamers had an advantage on processing speed and spatial reasoning performance relative to non-gamers. Among the recreational gamers, there was not a clear benefit of Action + or Puzzle + game play influence on cognitive performance. Adjusting for adolescent IQ further obscured these associations, with the advantageous gamer performance concentrated in spatial ability tasks. Sensitivity gamer-only analyses further confirmed these findings but revealed that adolescent IQ fully attenuated Puzzle + and speed associations and predicted the likelihood of adult Puzzle + game play preference. Spatial ability performance may benefit from video game engagement in general and may not be contingent on playing games with a specific gameplay mechanic such as ones that stem from Action + or Puzzle + game play. Even so, our findings suggest that pre-existing cognitive function plays an important role in adult gaming associations and highlights how underlying intrinsic factors may connect and contribute to later adult recreational selection.

In addressing our first aim to examine general video game play associations with cognitive ability, we found that being a gamer was significantly associated with better performance across all cognitive tasks, apart from the working memory task (i.e., Digit Span). These findings align with other cross-sectional study observations of the benefits of gaming for tasks that require information processing or visual spatial skills [58, 66]. We likely did not observe gaming associations with our sole working memory task because it lacks perceptual demands, a particular component required in playing most video games. This finding aligns with past work comparing gamer performance between different types of working memory tasks, such as those more reliant on visuospatial [87] or attentional [79] demands. To say another way, gamers may show higher performance on cognitive abilities that are directly represented and exercised in the games they play either through causal or reverse causation routes. Work from gaming training and intervention studies highlight the narrow cognitive transfer effect of gaming [72]. In such cases, improvements tend to be specific to certain tasks and may not generalize to broader cognitive domains. For example, while certain games may specifically train individuals on certain tasks, generalizable gains in improvement to other cognitive skills have not been well supported [72]. With respect to the magnitude of our effects, our gaming correlations were weakly associated with cognitive performance, ranging from 0.06 to 0.12 for processing speed and spatial ability, respectfully. While individual correlational studies have been found to vary widely in effect size, the meta-analytic r is more in line with our work ranging from 0.06 visual attention to 0.18 for spatial ability [66]. Likewise we found the mean differences between gamers and non-gamers were small in size (Cohen’s *d* = 0.17 to 0.25) after controlling for sociodemographics and digital screen time. Training and intervention studies are popular approaches in the gaming research field and our associations are smaller relative to the meta-analytic random effects found across cognitive abilities in quasi-experimental (Cohen’s *d* = 0.61) or true experimental (Cohen’s *d* = 0.48) studies, although effects by specific cognitive domains are generally smaller [58]. However, other meta-analytic work of training and intervention studies found weak evidence of a gaming effect across specific cognitive abilities [66]. Our work comes from a normative sample using naturalistic gaming assessments to measure gaming behavior, often differing from game-centric studies that recruit habitual gamers, and include detailed gaming metrics. There may be more blurred boundaries between our gamer and non-gamer distinction given we do not know gaming history, skill, or frequency and dichotomized metrics are known to lack granularity and power [30]. Nonetheless, even our study provides support that the gamer advantage can be uncovered in a large established adulthood sample.

Our study provides further insight in how earlier life cognitive ability may affect the robustness of findings within adult gaming research. Gaming-cognition associations were moderately attenuated, though remaining significant for all spatial reasoning tasks and one processing speed task, suggesting gaming effects are partially attributable to earlier life cognitive differences. Research has supported stronger gaming effects with spatial ability and theorized associations arose from complementary gaming mechanics playing may tap [7, 66]. Given the strength and consistency of prior findings from correlational to experimental studies [7, 58, 66], gaming-spatial cognition associations were expected post-adolescent IQ adjustment of the cognitive domains tested. It remains unclear why only one processing speed test, Colorado Perceptual Speed, remained significantly associated with gaming, but we speculate it may signal that the associated higher performance may be narrowly related to specific cognitive abilities rather than more generalizable within and between different cognitive domains. For example, this task may be more aligned with gaming mechanics that tap visual search and pattern recognition, unlike Subtraction and Multiplication. Interestingly, results for the Digit Symbol test differed from those of the Colorado Perceptual Speed test, which has similar perceptual demands but a greater motor component [46]. Some have argued that cognitive differences observed among gamers, especially for speed-based tasks, may arise due to enhancements to motor ability ([14, 26, 66, 81]). Our findings suggest otherwise, with the observed gaming associations remaining for the less physically demanding and dexterous processing speed task. In general, our findings align with gaming current literature while underscoring the need to account for underlying, and often unaccounted-for, intrinsic factors like earlier cognitive ability.

In addressing our second aim to further examine differential gameplay mechanic influences derived from Action + and Puzzle + video game play on cognitive ability, we observed limited evidence of a specific gameplay mechanic benefit across cognitive domains. In other words, while general gaming appeared to be associated with better cognitive performance, the specific mechanics that define the Action + (e.g., fast-paced gameplay) and Puzzle + (e.g., logical challenges) categories did not show as strong of an effect. Correlations supported Puzzle + and speed and Action + and spatial associations, which were not observed in the full analysis after controlling for sociodemographics and digital screen time. In gamer-only analyses, engagement with Puzzle + and Action + video games were associated with better Digit Symbol and Block Design performance, respectively. This difference may have emerged due to gameplay mechanic comparisons conducted in the sensitivity and main analyses. Gamer-only sensitivity analyses coded Action + and Puzzle + players as either playing games with core gameplay mechanics related to Action + and Puzzle + categories or not, whereas the main analyses directly contrasted Action + to Puzzle + exclusive players. Competing these gameplay mechanics derived from Action + and Puzzle + engagement in the main analyses may have obscured initial findings, especially for Action + and spatial associations, which have been robust in extant literature [7, 66].

Adults trained in action games have shown gains in processing speed tasks [86], differing from our findings. There are few studies that have examined processing speed, instead focusing on simpler perceptual and attentional tasks [7, 66]. Others have found puzzle-based enhancements in processing speed and reaction time among individuals who completed 30 rounds of Bejeweled Blitz [77] and task-switching abilities for individuals trained on commercial puzzle games [53]. The Digit Symbol subtest requires individuals to reproduce symbols and match them to their corresponding digit, leaving some to argue this test characteristic aligns with puzzle video gameplay that engages pattern recognition and sequence solving [8]. Our findings partially corroborate prior research, but gameplay mechanic effects were not domain consistent ––emerging for only a couple of tasks –– providing limited evidence for genre influence even before adolescent IQ adjustment.

Initially, we found limited evidence of a specific gameplay mechanic benefit, and post-adolescent IQ adjustment only revealed Action + players’ advantage over Puzzle + players. Sensitivity analyses further supported this finding with Action + players performing better relative to non-Action + players after controlling for pre-existing cognitive function. Beyond the video game play benefit on spatial abilities, it is surprising the added benefit of playing action games was seen with one spatial task considering how past work has emphasized this action genre [7, 40]. Perhaps we only replicated the action benefit with Block Design because this task requires 3-Dimensional (3-D) manipulation as opposed to the other two spatial tasks that inherently are 2-Dimensional in nature (e.g., Paper Form Board and Card Rotations). Many modern action games require navigating, interacting, and manipulating objects in 3-D simulated spaces, potentially highlighting practiced action gaming mechanics and strategies less transferrable to the spatial tasks (e.g., [15, 31]). Overall analyses suggest a particular saliency of action game play to the spatial Block Design task where benefits persisted even with the inclusion of earlier life general cognitive ability.

Next, cognitive matching may underlie Puzzle + and speed associations such that individuals may actively select or seek gameplay mechanics that match with their cognitive strengths. Puzzle + and speed connections found in sensitivity analyses exemplify a selection effect. Associations did not persist after the inclusion of adolescent IQ suggesting these behaviors are initially linked due to individual differences in cognitive ability rather than video game engagement. In other words, individuals who are more adept in their performance on IQ batteries may have a propensity for or are drawn to play Puzzle + games. Supporting this notion, adolescent IQ predicted the likelihood of playing Puzzle + video games but not Action + video games. Females were also more likely to play Puzzle + games and perform better on processing speed. The specific Puzzle + and speed findings may capture selection dynamics shared between sex, cognitive ability, and gaming preference on processing speed. Females did perform better on processing speed, replicating their known advantage within this domain [33]. How the interaction of sex and game engagement influences cognition beyond one’s pre-existing status remains to be further tested.

Our selection effect evidence aligns with prior literature, which found individuals with higher IQ scores tend to seek out more intellectually stimulating activities or more enriched environments that promote cognitive stimulation and cognitive development [61, 76]. Puzzle + games may tap skills related to strategic and abstract thinking, pattern recognition, problem solving, logical reasoning, and critical thinking skills, offering players a specified, targeted cognitive challenge to satisfy the game’s objective [37, 43]. Unlike Action + games, which can also tap these same cognitive abilities but place greater speed and perceptual demands on players, individuals may prefer puzzle games because of their preference for logical skill-based tasks [34, 69, 83]. Moreover, those with higher IQs often exhibit a strong inclination towards achieving mastery, a trait observed in educational contexts [19]. Future work would do well to examine how gamers approach Puzzle + games and whether further skill and mastery gains while playing, challenging their initial competence, contribute to cognitive functioning.

## Limitations

This study’s key strengths address gaps in gaming-cognition literature. Nonetheless, this study is not without limitations. One objective of CATSLife is understanding how distal and proximal lifestyle factors impact health and functioning [85]. With this broader study view, our measures of video game behavior do not capture the individual approach or the subjectivity surrounding gameplay [12] available in more specialized gaming studies. Our study compared the influence of gameplay mechanics, specifically derived from Action + and Puzzle + game play, on cognitive performance. However, comparing game genres is an imprecise method for contrasting gaming mechanics that may tap differing cognitive resources. Many gaming mechanics are not genre-exclusive with modern games blending mechanics between genres, making it difficult to isolate the effects of specific gaming mechanics [6, 13],. Additionally, our study relied on self-reported digital screen time and video game play, which, while valuable for capturing subjective experiences, may lack the validity of objective measures [94]. However, research has shown that subjective and objective measures of social media use have comparable predictive validity for outcomes such as well-being and self-esteem [45, 84].

Unlike many studies, we have the advantage of examining longitudinally informed cognitive measures. Yet, we lack a conclusive gaming history dating back to adolescence, and our present study is cross-sectional, limiting our ability to draw causal conclusions. Future research should consider how the age of onset for active video game play influences current cognitive performance. Longitudinal work is required to evaluate how gaming, and the onset of active video game play, may influence cognitive development, especially during established adulthood [52] when cognitive decline manifests for age-sensitive traits [42]. For example, adjusting for adolescent Block Design revealed potentially nuanced longitudinal trends. Unlike adjusting for adolescent IQ, which is age-invariant, adjusting for earlier Block Design, which fully attenuated all gaming effects, cannot capture the non-linear age trends known to exist across the lifespan [42, 50]. It remains to be seen if and how gaming influences cognitive status, or contributes to later cognitive growth and maintenance, similar to other cognitively and socially engaging leisure activities .

## Conclusion

Our work provides further support for positive associations between gaming and cognition, specifically spatial ability. Moreover, the benefits are not restricted to playing a certain core gameplay mechanic derived from Action + or Puzzle + game play, especially ones favored by younger men. To date, gaming-cognition literature has lacked female and established adult representation or measures to account for reverse causation (c.f., [8, 52]). Our study directly tackles those key concerns to understand adult gaming-cognition associations and extends current knowledge on how earlier cognitive function informs those links. Individual differences in cognitive performance contribute to recreational genre preference, predominately among Puzzle + players. Specific gameplay mechanic benefits only emerged for the relationship between Action + and spatial ability, whether and how differential gameplay mechanic engagement contributes to later cognitive aging will be necessary future work. With the increasing sophistication and complexity of video games, their potential as an intervention tool for enhancing or maintaining cognitive functioning holds great future potential.

## Supplementary Information

Additional file 1.

## Data Availability

For inquiries regarding access to the data presented in the current study, contact the corresponding author. Requests for data, except where participant directives and IRB directives do not permit us to do so, will require the completion of a data use agreement, documentation of training in the protection of human subjects, and the purpose of use.

## Footnotes

1 Additional details on the genre coding and familiarity rating among coders may be found in our codebook available on OSF: https://osf.io/z2wq8/?view_only=18151e479419497fae8c3d2053ef13a8

2 Post-hoc genre analysis adjusting for adolescent BD, subsequently attenuated the VGplay effect leaving associations trending on significance (B = 0.75, p = 0.07, see Supplemental Table ST 5). These patterns may suggest more nuanced longitudinal contributions shaping BD performance and VGplay.
